# Ambulatory Risk Models for the Long-Term Prevention of Sepsis: Retrospective Study

**DOI:** 10.2196/29986

**Published:** 2021-07-08

**Authors:** Jewel Y Lee, Sevda Molani, Chen Fang, Kathleen Jade, D Shane O'Mahony, Sergey A Kornilov, Lindsay T Mico, Jennifer J Hadlock

**Affiliations:** 1 Institute for Systems Biology Seattle, WA United States; 2 Swedish Center for Research and Innovation Swedish Medical Center Seattle, WA United States; 3 Providence St Joseph Health Renton, WA United States

**Keywords:** sepsis, machine learning, electronic health records, risk prediction, clinical decision making, prevention, risk factors

## Abstract

**Background:**

Sepsis is a life-threatening condition that can rapidly lead to organ damage and death. Existing risk scores predict outcomes for patients who have already become acutely ill.

**Objective:**

We aimed to develop a model for identifying patients at risk of getting sepsis within 2 years in order to support the reduction of sepsis morbidity and mortality.

**Methods:**

Machine learning was applied to 2,683,049 electronic health records (EHRs) with over 64 million encounters across five states to develop models for predicting a patient’s risk of getting sepsis within 2 years. Features were selected to be easily obtainable from a patient’s chart in real time during ambulatory encounters.

**Results:**

The models showed consistent prediction scores, with the highest area under the receiver operating characteristic curve of 0.82 and a positive likelihood ratio of 2.9 achieved with gradient boosting on all features combined. Predictive features included age, sex, ethnicity, average ambulatory heart rate, standard deviation of BMI, and the number of prior medical conditions and procedures. The findings identified both known and potential new risk factors for long-term sepsis. Model variations also illustrated trade-offs between incrementally higher accuracy, implementability, and interpretability.

**Conclusions:**

Accurate implementable models were developed to predict the 2-year risk of sepsis, using EHR data that is easy to obtain from ambulatory encounters. These results help advance the understanding of sepsis and provide a foundation for future trials of risk-informed preventive care.

## Introduction

Sepsis is a life-threatening condition characterized by a systemic immunological response to infection. Each year, more than 1.7 million adults in the United States develop sepsis, and nearly 16% of them die [1]. It is the leading cause of death in hospitals worldwide and puts a huge burden on health care systems [2-4]. Research to date has primarily focused on the inpatient setting, where timely treatment can improve sepsis-associated mortality and morbidity [5-9]. Commonly used risk scores, such as the systemic inflammatory response syndrome (SIRS) score [10], quick sequential organ failure assessment (qSOFA) score [11], and modified early warning score (MEWS) [12], offer benefit once patients are acutely ill, but are less useful for early detection [13-16]. Advanced machine learning has led to more efficient models based on data from larger populations and a greater number of risk factors [17-21], but these are designed for emergency and inpatient settings [21-27].

Better risk models are needed to support community-acquired sepsis prevention. In 2016, Wang et al were the first to develop a risk score for long-term sepsis [28]. Using the REGARDS cohort (n=30,239), they predicted an individual’s 10-year risk of sepsis (REGARD SRS), with a bootstrapped C index of 0.703. The REGARD SRS and SSRS rely on demographic and medical history features that could be obtained by patient self-report, but they also depend on clinical laboratory results from blood and urine, including laboratory tests, such as cystatin-C and high-sensitivity C-reactive protein, which are not routinely measured in community-dwelling patients. Thus, there is a pressing need for a noninvasive solution to guide interventions for preventing sepsis, including immunization, education on infection prevention, and early symptom recognition [29,30]. Published guidelines currently recommend these interventions for some patients, such as those who will be experiencing neutropenia secondary to chemotherapy or posttransplant immunosuppression [31,32], but many other patients at high risk are overlooked. An implementable model that works on real-world patient data could support risk stratification for population health outreach or at the point of care.

Given the increased adoption of electronic health records (EHRs) in ambulatory care [33], a wealth of longitudinal phenotype and exposure data is now accessible to support predictive analytics. Sepsis risk research can move beyond inpatient encounters toward investigation of long-term patient trajectories. Historical data can support more accurate models for clinical decision support and improved resource stewardship. Yet, accuracy is only one dimension of model quality. Two other considerations are implementability in real-world settings and biomedical relevance for discovery of new hypotheses about the mechanisms of disease, prevention, and treatment.

In this study, we developed EHR-based models using supervised machine learning methods to predict the long-term risk of sepsis, investigating both time-invariant and temporal synopsis features. For each model, we reported results for both performance and feature importance, and discussed trade-offs between accuracy, interpretability, implementability, and biomedical relevance. This research investigated the potential to predict long-term sepsis risk in ways that can inform clinical decisions and lead to a better understanding of the disease.

## Methods

### Data and Study Setting

Providence St. Joseph Health (PSJH) is a community health system that includes over 51 hospitals and 1085 clinics. This retrospective study used clinical data from PSJH EHRs for patients who presented for health care at Providence, Swedish, or Kadlec sites in Alaska, California, Montana, Oregon, and Washington. Research was conducted within a Health Insurance Portability and Accountability Act (HIPAA)-secure data platform, after date shifting had been applied to reduce the risk of reidentification. Dates were shifted using a randomly selected offset per patient of up to ±365 days. All time windows below were defined on postshifted dates. Procedures were approved by the Institutional Review Board (IRB) at PSJH (IRB Study Number STUDY2019000389). Records were included for patients who presented for health care at least one time between 2017 and 2019. Our prediction model used records from patients over 18 years of age during a 3-year observation window starting in 2014 to predict sepsis in a 2-year window, starting in 2017. Patient age was calculated for the prediction window start date. Patients with no valid birth date or no encounters prior to 2014 were excluded. Our final study cohort consisted of 2,683,049 patients, including 1,558,851 (58.1%) women and 1,124,198 (41.9%) men, and the median age was 51.36 years. Over 64,000,000 encounters were collected from the cohort patients for feature extraction.

### Feature and Label Extraction

Features represent information about the data used as model inputs, and the label is the outcome that the model is trained to predict. In this study, we selected features that can be easily obtained from EHRs, including previously reported long-term risk factors for sepsis [34] and potential risk factors for investigation. Binary outcome variables were used in labeling for classification (1 for sepsis and 0 for no sepsis). Sepsis was defined using the Systematized Nomenclature of Medicine-Clinical Terms (SNOMED CT) [35] hierarchical terminology system. The label was set to 1 if the parent concept for sepsis, SNOMED CT identifier (SCTID = 91302008), or any of its descendants was found in the problem list during the prediction window.

The following features were extracted from the observation window: sex, age, ethnicity, race, height, weight, BMI, ambulatory vital signs, history of medical conditions, hospital length of stay, encounters, problem list entries, medical history entries, medication orders, and procedures. Medical conditions were considered present if the SNOMED CT parent concept or any of its descendant concepts were found in the problem list during the observation window. The sepsis feature was included to investigate whether having a history of sepsis is a risk factor for developing sepsis in the future. Ratio features with repeated observations (eg, BMI, vital signs, and hospital length of stay) were transformed through statistical aggregation (minimum, maximum, mean, and standard deviation). All features are defined in Table 1 and categorized into four feature sets as follows: basic, vital signs, medical history, and health care delivery data. In total, 49 features were entered into the supervised machine learning process.

**Table 1:** Definitions of features used for models in the study for the observation window.

Category		Definition
**Basic features**		
	Sex	Male (1), female (0), missing (−1)
	Age	Age calculated at the start of the prediction window
	Race	Native Hawaiian/Pacific Islander, American Indian/Alaska Native, Asian, Black/African American (1); White (0); other/missing (−1)
	Ethnicity	Hispanic/Latino (1), not Hispanic/Latino (0), missing (−1)
	Height	Last observed height
	Weight	Last observed weight
	Std_BMI	Standard deviation of BMI
**Vital sign features**		
	BP_sys	Average and standard deviation of systolic blood pressure
	BP_dia	Average and standard deviation of diastolic blood pressure
	BT	Average and standard deviation of body temperature
	HR	Average and standard deviation of heart rate
	RR	Average and standard deviation of respiratory rate
**Medical history features**		
	Sepsis	Sepsis (SCTID^a^ 91302008)
	Pneumonia	Pneumonia (SCTID 233604007)
	Bacterial infection	Bacterial infectious disease (SCTID 87628006)
	Fungal infection	Mycosis (SCTID 3218000)
	Protein-energy malnutrition	Deficiency of macronutrients (SCTID 238107002)
	Cancer	Malignant neoplastic disease (SCTID 363346000)
	COPD^b^	Chronic obstructive lung disease (SCTID 13645005)
	Diabetes	Diabetes mellitus (SCTID 73211009)
	Chronic kidney disease	Chronic kidney disease (SCTID 709044004)
	Hypertension	Hypertensive disorder, systemic arterial (SCTID 38341003)
	Deep vein thrombosis	Deep venous thrombosis (SCTID 128053003)
	Arteriosclerosis	Arteriosclerotic vascular disease (SCTID 72092001)
	Peripheral artery disease	Peripheral arterial occlusive disease (SCTID 399957001)
	Coronary artery disease	Coronary arteriosclerosis (SCTID 53741008)
	Heart attack	Myocardial infarction (SCTID 22298006)
	Atrial fibrillation	Atrial fibrillation (SCTID 49436004)
	Stroke	Cerebrovascular accident (SCTID 230690007)
	Heart failure	Heart failure (SCTID 84114007)
**Health care delivery features**		
	n_encounter	Total count of clinical encounters
	n_hospitalization	Total count of hospitalizations
	LOS	Average, minimum, maximum, and standard deviation of length of hospital stay
	n_problem	Total count of problem list entries
	u_problem	Number of unique problem list entries
	n_medical_hx	Total count of medical history entries
	u_medical_hx	Number of unique medical history entries
	n_medication	Total count of prescription medication orders
	u_medication	Number of unique prescription medication orders
	n_procedure	Total count of ordered medical procedures
	u_procedure	Number of unique ordered medical procedures

^a^SCTID: Systematized Nomenclature of Medicine-Clinical Terms (SNOMED CT) identifier.

^b^COPD: chronic obstructive pulmonary disease.

### Machine Learning

Data preprocessing and cleaning were conducted as follows. Missing data in categorical features (sex, race, and ethnicity) were assigned to be −1. Missing data in height, weight, and vital signs were imputed using the carry-forward method if previous observations were available; otherwise, median imputation was used. Outliers in height and weight were detected by calculating the modified z-score based on median absolute deviation (MAD) [36] in equation 1 with a threshold of 3.5. Both outliers and missing data were imputed with the median. Equation 1 is as follows:

Mi = 0.6745 (xi−x̃) MAD (**1**)

where MAD is the median absolute deviation and x̃ is the median of x.

**Figure 1:**
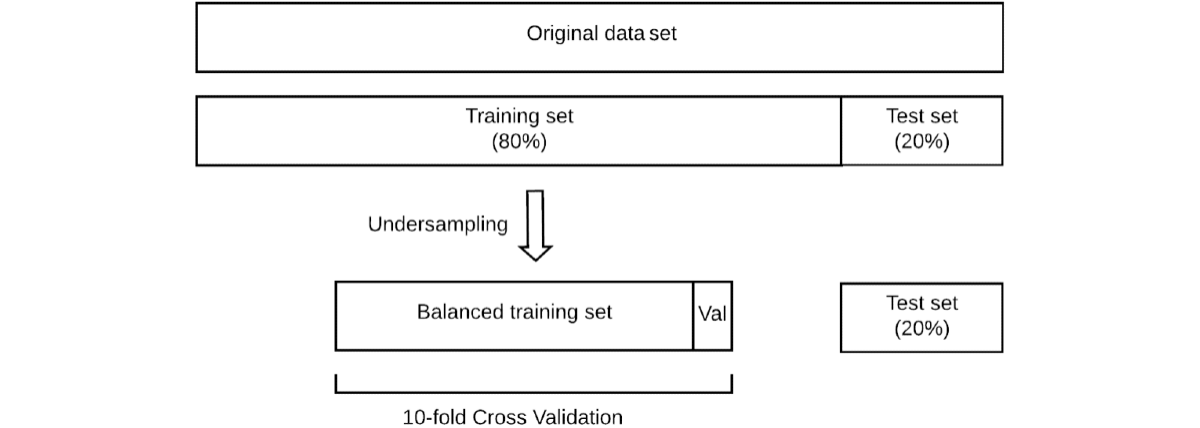
Training, validation, and test split for modeling of the long-term risk of sepsis.

Patients diagnosed with sepsis accounted for only about 0.8% of the cohort, leading to extremely imbalanced data. To ensure the validity of the model but, at the same time, overcome the class imbalance in the medical data set, we reserved 20% of the original data as a test set and undersampled the other 80% of the data by randomly selecting the same number of patients from the majority class (no sepsis) as the minority class (sepsis) to construct a balanced training set. The train/test split process is shown in Figure 1. This training set was then trained with several machine learning methods, including gradient boosting (GB), support vector machine (SVM), and logistic regression (LR), and validated with 10-fold cross validation. Four models were constructed with different combinations of feature sets. Model 1 used only the basic features. Sequentially, we added vital sign features to model 2, medical history features to model 3, and health care delivery data features to model 4.

### Model Performance Evaluation

All classification models were built using scikit-learn [37], an open-source Python machine learning library. Widely adopted performance measures, such as area under the receiver operating characteristic curve (AUROC), precision, sensitivity (or recall), specificity, and likelihood ratio, were used to evaluate the discrimination ability of our prediction models. Appropriate measures were selected based on the class distribution in the models. We also analyzed relative feature importance using the following three methods: (1) Shapley Additive exPlanations (SHAP) algorithm, (2) permutation testing, and (3) model coefficients from L1-regularized logistic regression (L1-LR). SHAP, an algorithm developed from coalition game theory, calculates the average marginal contribution of a feature across all possible coalitions [38]. Permutation testing estimates feature importance by calculating the drop in the performance after permuting the feature. A feature is considered important if shuffling its values increases the model prediction error. Shapley values and permutation feature importance computed on test data avoid the systematic bias in feature selection found with mean decrease impurity–based measures [39]. We also retrieved coefficients from L1-LR to investigate the relevance and directionality of features. LR with L1 regularization is a sparse linear model in which coefficients for unimportant features are reduced to zero [40], and the sign of the coefficient suggests positive or negative association with the model outcome (sepsis) [41].

## Results

Table 2 shows the results of 10-fold cross-validation based on training data using GB, SVM, and LR. The results show a consistent trend of model performance, increasing as more features were added. GB slightly outperformed linear classifiers (SVM and LR) in all four models. The best AUROC of 0.8216 was achieved by model 4. The trained GB models were then used to make predictions on the 20% test data set, and they were evaluated with precision, sensitivity (or recall), specificity, positive and negative likelihood ratios, and diagnostic odds ratios because of the highly imbalanced class distribution (Table 3). The test set prevalence was 0.0079 with the population size of 536610. The results showed that the positive likelihood ratio ranged from 2.1135 to 2.8897, and the negative likelihood ratio ranged from 0.3192 to 0.4997. Sensitivity and specificity in each model had similar results in the training set and test set for predicting the sepsis outcome.

**Table 2:** Ten-fold cross-validation results on the training set.

Model and classifier		Precision	Sensitivity	Specificity	AUROC^a^	Ten-fold error (%)
**Model 1 (basic)**						
	GB^b^	0.6727	0.6725	0.6725	0.7349	0.29%
	SVM^c^	0.6607	0.6606	0.6606	0.7167	0.27%
	LR^d^	0.6569	0.6565	0.6565	0.7134	0.29%
**Model 2 (basic + VS^e^)**						
	GB	0.6947	0.6946	0.6946	0.7595	0.28%
	SVM	0.6812	0.6811	0.6811	0.7425	0.29%
	LR	0.6776	0.6775	0.6775	0.7399	0.26%
**Model 3 (basic + VS + MHX^f^)**						
	GB	0.7008	0.7006	0.7006	0.7671	0.20%
	SVM	0.6897	0.6868	0.6868	0.7502	0.17%
	LR	0.6893	0.6891	0.6891	0.7523	0.18%
**Model 4 (basic + VS + MHX + HCD^g^)**						
	GB	0.7483	0.7481	0.7481	0.8216	0.27%
	SVM	0.7191	0.7169	0.7169	0.7910	0.26%
	LR	0.7185	0.7175	0.7175	0.7835	0.19%

^a^AUROC: area under the receiver operating characteristic curve.

^b^GB: gradient boosting.

^c^SVM: support vector machine.

^d^LR: logistic regression.

^e^VS: vital signs.

^f^MHX: medical history.

^g^HCD: health care delivery data.

**Table 3:** Prediction results and 95% confidence intervals for the test set using the trained gradient boosting model.

Model	Precision, value (95% CI)	Sensitivity, value (95% CI)	Specificity, value (95% CI)	LR+^a^, value (95% CI)	LR−^b^, value (95% CI)	DOR^c^
Model 1 (basic)	0.0165 (0.0159-0.0171)	0.6552 (0.6407-0.6694)	0.6900 (0.6887-0.6912)	2.1135 (2.0670-2.1611)	0.4997 (0.4793-0.5209)	4
Model 2 (basic + VS^d^)	0.0177 (0.0171-0.0184)	0.6862 (0.6721-0.7001)	0.6980 (0.6968-0.6993)	2.2724 (2.2256-2.3202)	0.4495 (0.4299-0.4701)	5
Model 3 (basic + VS + MHX^e^)	0.0184 (0.0177-0.0190)	0.6874 (0.6733-0.7012)	0.7084 (0.7071-0.7096)	2.3570 (2.3086-2.4065)	0.4413 (0.4220-0.4615)	5
Model 4 (basic + VS + MHX + HCD^f^)	0.0224 (0.0217-0.0231)	0.7653 (0.7523-0.7779)	0.7352 (0.7340-0.7363)	2.8897 (2.8401-2.9401)	0.3192 (0.3023-0.3371)	9

^a^LR+: positive likelihood ratio.

^b^LR−: negative likelihood ratio.

^c^DOR: diagnostic odds ratio.

^d^VS: vital signs.

^e^MHX: medical history.

^f^HCD: health care delivery data.

**Figure 2:**
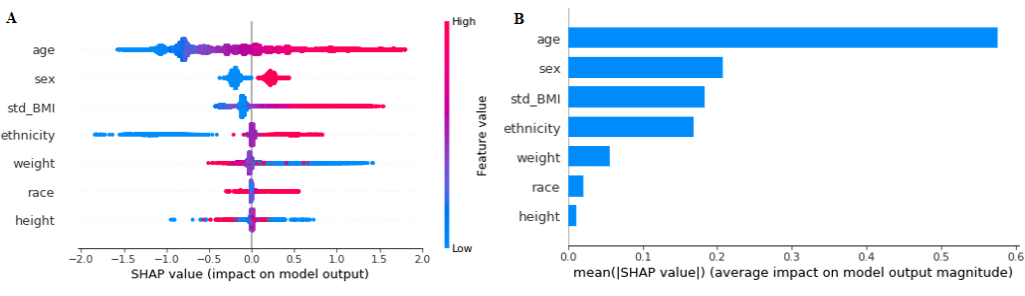
The Shapley Additive exPlanations (SHAP) algorithm results for long-term sepsis risk in model 1. (A) The influence of higher and lower values of the feature on the patient's outcome. The left side of this graph represents reduced risk of developing sepsis, and the right side of the graph represents increased risk of developing sepsis. Red dots represent higher values of the feature, and blue dots represent lower values of the feature. Nominal classes are binary (0,1). (B) The ranking of feature importance indicated by SHAP.

**Figure 3:**
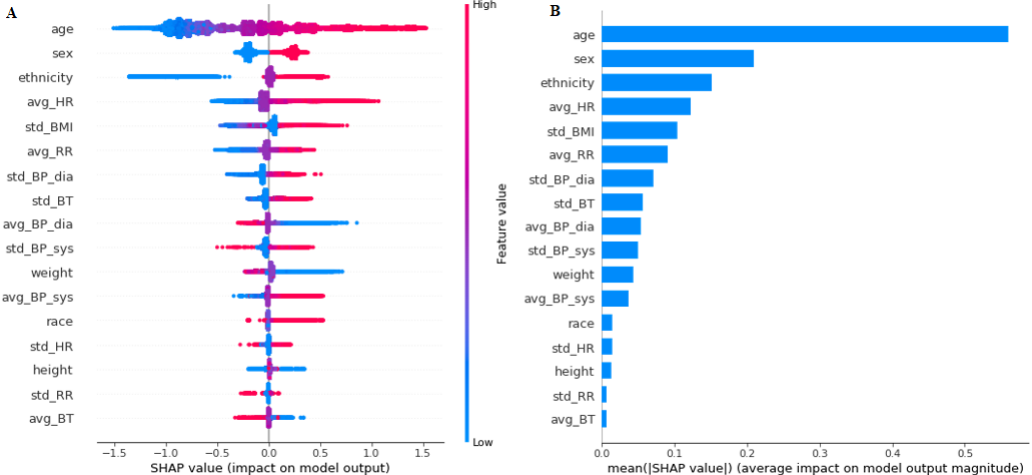
The Shapley Additive exPlanations (SHAP) algorithm results for long-term sepsis risk in model 2. (A) The influence of higher and lower values of the feature on the patient's outcome. The left side of this graph represents reduced risk of developing sepsis, and the right side of the graph represents increased risk of developing sepsis. Red dots represent higher values of the feature, and blue dots represent lower values of the feature. Nominal classes are binary (0,1). (B) The ranking of feature importance indicated by SHAP. BP: blood pressure; BT: body temperature; HR: heart rate; RR: respiratory rate.

**Figure 4:**
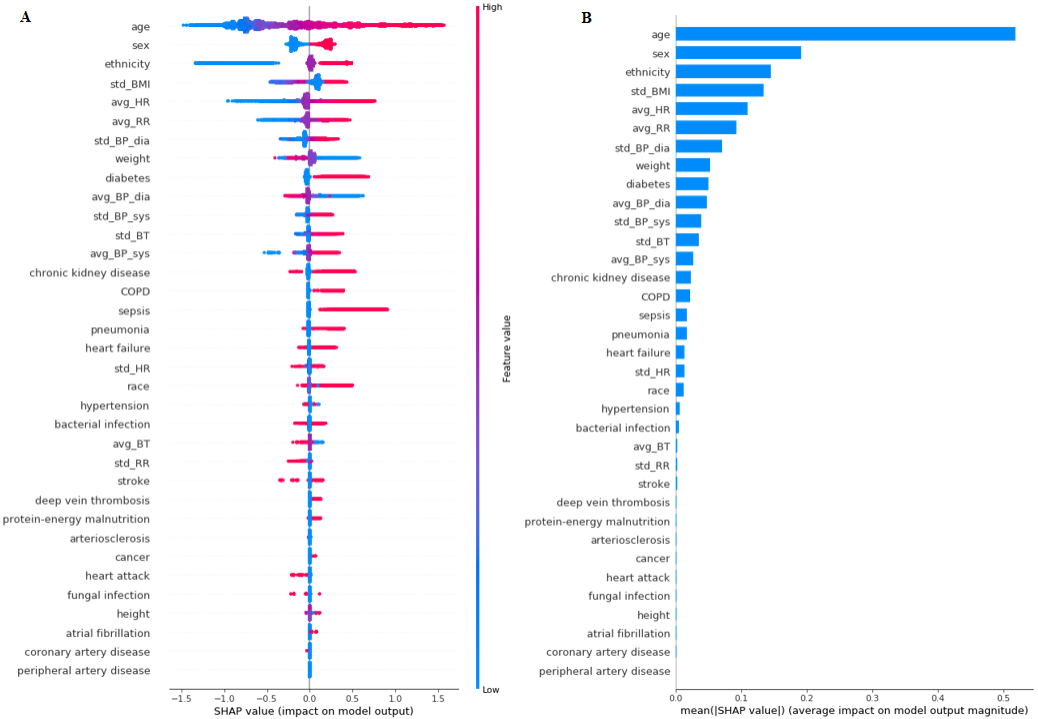
The Shapley Additive exPlanations (SHAP) algorithm results for long-term sepsis risk in model 3. (A) The influence of higher and lower values of the feature on the patient's outcome. The left side of this graph represents reduced risk of developing sepsis, and the right side of the graph represents increased risk of developing sepsis. Red dots represent higher values of the feature, and blue dots represent lower values of the feature. Nominal classes are binary (0,1). (B) The ranking of feature importance indicated by SHAP. BP: blood pressure; BT: body temperature; COPD: chronic obstructive pulmonary disease; HR: heart rate; RR: respiratory rate.

**Figure 5:**
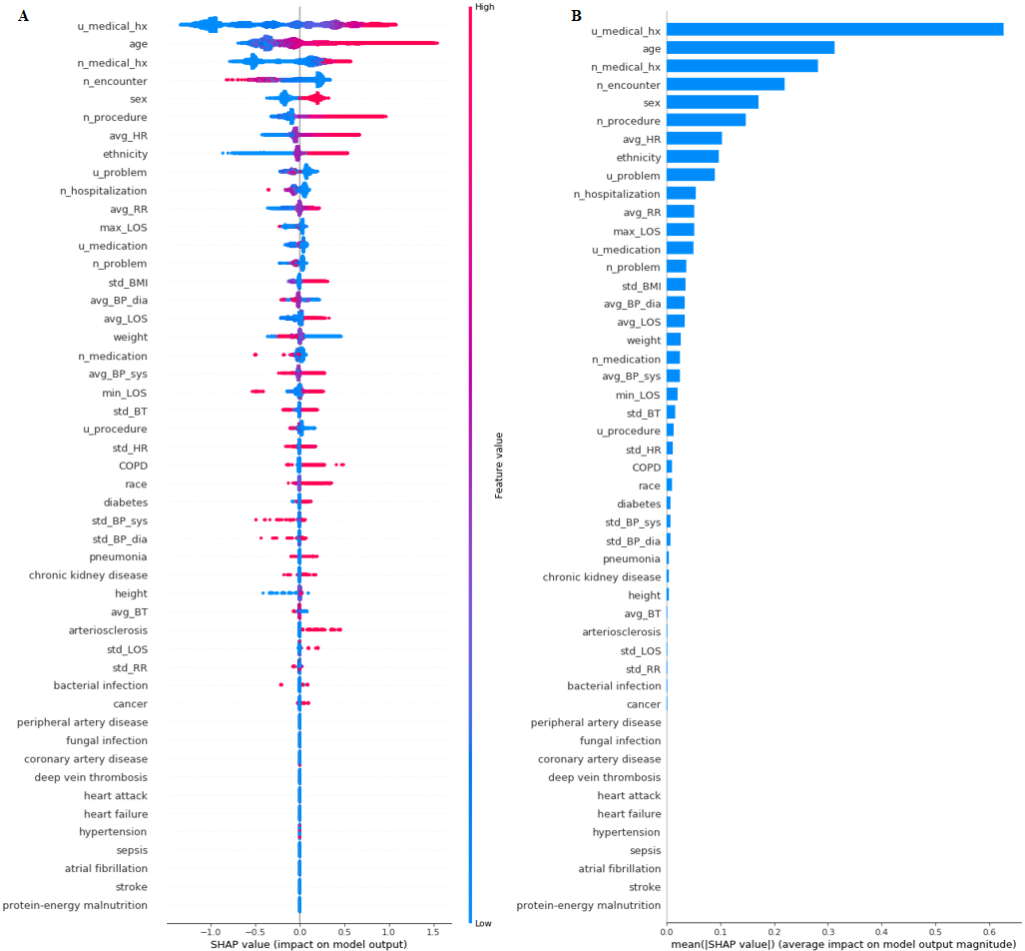
The Shapley Additive exPlanations (SHAP) algorithm results for long-term sepsis risk in model 4. (A) The influence of higher and lower values of the feature on the patient's outcome. The left side of this graph represents reduced risk of developing sepsis, and the right side of the graph represents increased risk of developing sepsis. Red dots represent higher values of the feature, and blue dots represent lower values of the feature. Nominal classes are binary (0,1). (B) The ranking of feature importance indicated by SHAP. BP: blood pressure; BT: body temperature; COPD: chronic obstructive pulmonary disease; HR: heart rate; LOS: length of hospital stay; RR: respiratory rate.

**Figure 6:**
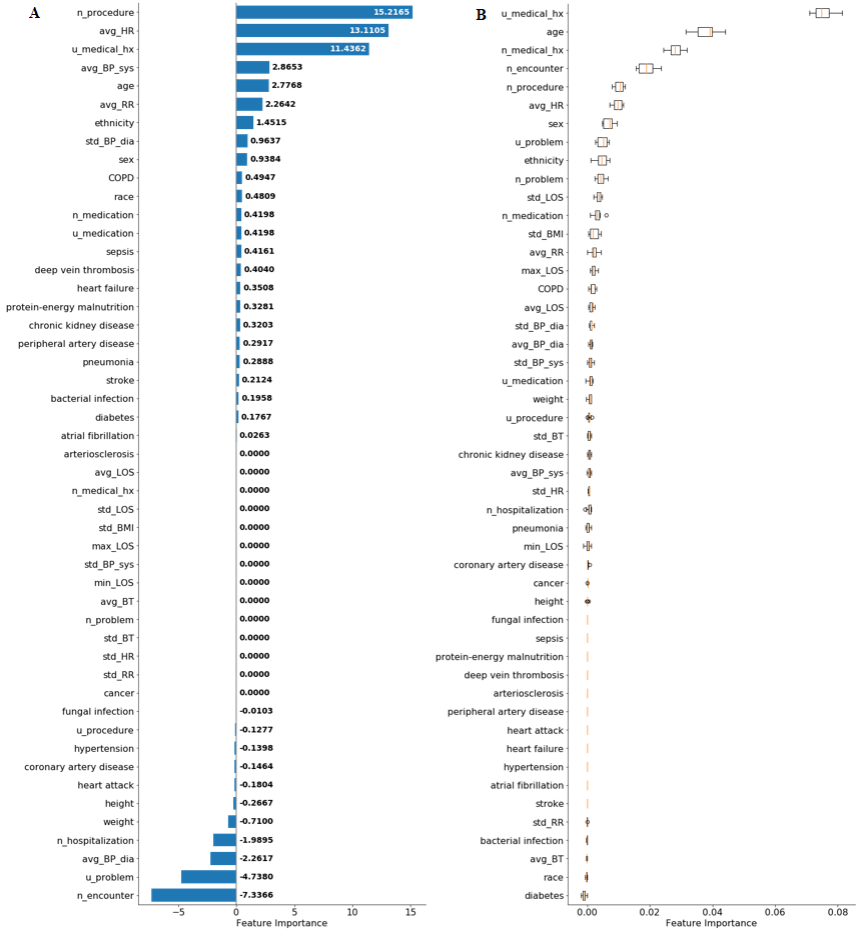
The L1-regularized logistic regression (L1-LR) algorithm results (A) and permutation testing results for long-term sepsis risk in model 4 (B). BP: blood pressure; BT: body temperature; COPD: chronic obstructive pulmonary disease; HR: heart rate; LOS: length of hospital stay; RR: respiratory rate.

To ensure the stability and reliability of the model, SHAP and permutation testing methods were implemented on the GB model. These methods improve the interpretability of the black box model and give a reasonable explanation for the prediction of each outcome. The results for the SHAP algorithm are shown in Figures 2-5. In addition, L1-LR and permutation results for model 4 are presented in Figure 6. In models 1-3, where health care delivery data features were not used, SHAP showed age as the dominant feature for predicting sepsis. Other important features included sex, ethnicity, respiratory rate, heart rate, standard deviation of BMI, history of sepsis, diabetes, and chronic kidney disease. In model 4, where health care delivery data features were added, the most predictive features were the number of unique entries (u_medical_hx), followed by age, the total count of medical history entries (n_medical_hx), the total count of encounters (n_encounter), sex, and the total count of ordered medical procedures (n_procedure). The important features identified in the SHAP algorithm have high permutation importance and high absolute values of coefficients learned by L1-LR models. The sign of the coefficients showed the directionality of those features. Moreover, the average diastolic blood pressure (avg_BP_dia) and the total count of encounters (n_encounter) were assigned with a negative coefficient in all three models, which implied the effect of high values for these features in decreasing the risk of developing sepsis.

## Discussion

### Principal Findings

In this study, we constructed four interpretable implementable EHR-based models to predict the 2-year risk of sepsis in adults. Each model performed well, considering the complexity of the features included. As expected, model 4 with all 49 features outperformed the others, with an AUROC of 0.8216 achieved by the GB algorithm in the training set. Due to the low prevalence of sepsis outcomes in the 20% test set, the precision was low in all models. However, the positive likelihood ratio of 2.8897 and negative likelihood ratio of 0.3192 achieved by model 4 showed that our model has the ability to identify patients with higher risk of sepsis. The dominant features in this model, accounting for more than half of the feature importance, were the numbers of unique and total medical history entries (u_medical_hx and n_medical_hx), and age. Medical history features suggest an increased burden of underlying health conditions, and aging is the most substantial risk factor for multimorbidity [42]. Comorbidities are known to be significantly higher in patients with sepsis compared to those without sepsis [1,43], but previous models have not included multimorbidity as a distinct feature. Another strong predictor in model 4 was the total number of ordered medical procedures (n_procedure). Procedures, particularly those that are invasive, increase the risk of hospital-acquired infections, and may also be indicative of health status and multimorbidity. The total number of encounters (n_encounter), which was assigned a negative coefficient in L1-LR, was also a strong predictor in model 4. Although it requires further investigation, one possible reason could be that a greater number of health care visits is associated with better access to preventative health care.

Age, ethnicity, sex, average heart rate (avg_HR), and standard deviation of BMI (std_BMI) were the most important features in models 2 and 3. In addition to increasing the risk of multimorbidity, age is a known independent risk factor for sepsis incidence, severity, and outcomes [44]. Whether ethnicity represents a sepsis risk factor is not yet established. Results from epidemiological studies are contrasting [45-47]. Ethnicity may also be associated with socioeconomic status, a health determinant recently found to be associated with a higher rate of hospital admissions for infection [48]. Future tracking of health-related social needs in structured EHR data [49] will support deeper investigation. A higher resting heart rate, which is common in infection, is also a risk factor for all-cause mortality [50] and may suggest a poorer health status. Patients with higher average heart rates may have had infections during previous encounters. Obesity and malnourishment are known risk factors for sepsis [51], but the standard deviation of BMI (change over time) is a new potential risk factor and merits investigation. In models 3 and 4, basic features and vital signs (age, ethnicity, sex, BMI, and heart rate) appeared to be more stronger predictors than well-established medical conditions known to be sepsis risk factors, including heart failure [52], chronic kidney disease [53], chronic obstructive pulmonary disease (COPD) [54], and diabetes [55,56]. Taken together, these findings suggest the possibility that sepsis risk is associated with not only age and medical conditions, but also vital signs and features related to health care delivery.

Although the highest performance was achieved with the health care delivery data features set, it has limited usefulness for discovering potential risk factors given its reliance on aggregated features, such as the number of medical history entries. Inclusion of these aggregated features weakens other predictors that are potentially more biomedically informative, including medical conditions and biomarkers, such as vital signs. The second-best performing model (model 3) identified a subset of biomarkers as strong predictors, including the standard deviation of BMI and average resting heart rate.

In models 3 and 4 that incorporated medical history, the conditions with greater importance for long-term sepsis risk were history of sepsis, heart failure, chronic kidney disease, pneumonia, COPD, and diabetes. In contrast, the most impactful chronic diseases in the REGARDS 10-year prediction score were chronic lung disease, followed by diabetes and peripheral artery disease [28,34]. The difference in risk factors between REGARDS and our models may reflect a different population sample and prediction window, but could also reflect differing definitions for conditions. For example, Wang et al used laboratory markers (estimated glomerular filtration rate, urinary albumin-to-creatinine ratio, and cystatin-C) for chronic kidney disease [28]. We selected diagnostic codes, which are less precise, but more likely to be consistently implementable on EHR data. SNOMED CT was selected because it is a medically curated semantic ontology, which is structured as a directed acyclic graph and used in EHRs across many countries. These codes can be mapped to ICD-10 codes, but different health care systems would likely benefit from retraining and retesting the model for their specific population.

The primary goal of this study was to investigate whether readily available EHR data can predict the long-term risk of developing sepsis during ambulatory visits in real time. Performance could also be useful for assessing population health. Interpretability was a secondary concern, and the feature importance estimates discussed above should be taken as exploratory. Relationships identified in the models reflected shared information content, but not necessarily biomedical relevance or causality. However, feature importance models suggested new insights on potential risk factors for sepsis that merit further investigation.

### Limitations

The studied population may have sample bias toward patients with continuous care within one health care system. There are also many common issues with structured EHR data that hamper the extraction of accurate information, including missing data, erroneous data, differences in EHR conventions among providers, and changes in how data are stored in EHRs over time [57]. These were only partially offset by terminology mapping, data removal, or imputation.

Using EHR diagnostic codes to identify sepsis patients also has limitations. First, it may miss cases where patients had sepsis at a different health care system. Second, because there is no confirmatory diagnostic test for sepsis, this model included patients who were treated empirically for sepsis but might not have had it. Third, variations in sepsis diagnosis, documentation, and coding practices could lead to missing sepsis labels [58]. Fourth, it does not differentiate between severe and milder forms of sepsis, or between hospital-acquired and community-acquired sepsis [43].

Future models can take advantage of the Adult Sepsis Event surveillance definition optimized for EHRs, which was recently released by the CDC [1,59]. This criterion uses objective clinical data to identify severe sepsis in hospitalized patients and displays superior sensitivity to diagnostic codes [1]. Lastly, our definition of ambulatory vital signs may include those that were taken in urgent or emergency situations. This is valid for prediction on real-world EHR data, but future models could better distinguish urgent encounters from those that are more likely to represent outpatient baseline.

### Conclusions

Strategies for long-term sepsis risk prediction are needed to advance the understanding of the disease and guide efforts for prevention. We used retrospective EHR data from 2,683,049 adults across five US states to develop models for predicting adult patients’ long-term risk of sepsis. Our models achieved a high AUROC and suggested new insights into potential long-term risk factors, including changes in BMI and a higher mean heart rate in ambulatory settings. These models could be implemented at a low cost, requiring only information that is easy to obtain from EHRs in real time. Ambulatory patients at the highest risk for sepsis could benefit from personalized preventative approaches, including increased emphasis on immunization, and education on reducing the risk of infection and recognizing early symptoms of sepsis. This implementable model provides a path toward clinical trials of risk-informed interventions for long-term sepsis prevention.

## Abbreviations

AUROC: area under the receiver operating characteristic curve
COPD: chronic obstructive pulmonary disease
EHR: electronic health record
GB: gradient boosting
L1-LR: L1-regularized logistic regression
LR: logistic regression
MAD: median absolute deviation
PSJH: Providence St. Joseph Health
SCTID: Systematized Nomenclature of Medicine-Clinical Terms identifier
SHAP: Shapley Additive exPlanations
SNOMED CT: Systematized Nomenclature of Medicine-Clinical Terms
SVM: support vector machine
